# Comparing Different Multimodal Analgesia Protocols for Primary Total Knee Arthroplasty—A Retrospective Cohort Analysis

**DOI:** 10.3390/jcm13144079

**Published:** 2024-07-12

**Authors:** Otto Koczian, Harald Winkler, Nelly Zental, Moritz M. Innmann, Fabian Westhauser, Tilman Walker, Dania Fischer, Markus A. Weigand, Sebastian O. Decker

**Affiliations:** 1 Medical Faculty Heidelberg, Department of Anesthesiology, Heidelberg University, Im Neuenheimer Feld 420, 69120 Heidelberg, Germany; otto.koczian@med.uni-heidelberg.de (O.K.); harald.winkler@med.uni-heidelberg.de (H.W.); nelly.zental@med.uni-heidelberg.de (N.Z.); dania.fischer@med.uni-heidelberg.de (D.F.); markus.weigand@med.uni-heidelberg.de (M.A.W.); 2 Medical Faculty Heidelberg, Department of Orthopedics, Heidelberg University, Schlierbacher Landstrasse 200A, 69118 Heidelberg, Germany; moritz.innmann@med.uni-heidelberg.de (M.M.I.); fabian.westhauser@med.uni-heidelberg.de (F.W.); tilman.walker@med.uni-heidelberg.de (T.W.)

**Keywords:** infiltration between the popliteal artery and capsule of the posterior knee (IPACK), single adductor canal block (SACB), total knee arthroplasty (TKA), femoral nerve block (FNB), popliteal sciatic nerve block (PSB)

## Abstract

**Background:** Several local regional anesthesia regimes have been described in the literature to reduce post-surgical pain following total knee arthroplasty (TKA), but it is unclear which regime has the best analgetic effect combined with the best motor function. The aim of this study was to determine if patients with infiltration between the popliteal artery and capsule of the posterior knee (IPACK) combined with an adductor canal block (SACB) had less pain, better motor function, and less opioid consumption after TKA than patients with a femoral nerve block (FNB) combined with a popliteal sciatic nerve block (PSB). **Methods:** In a retrospective cohort analysis, 342 patients following primary TKA were examined; 175 patients were treated with an IPACK combined with a SACB, and 167 patients with a femoral FNB combined with a PSB. The outcome parameters postoperative pain (visual analogue scale (VAS) for mobilization and at rest, functional recovery, opioid consumption, hospital discharge, and complications were analyzed and compared between both groups. **Results:** The IPACK/SACB group had a higher postoperative need for opioids despite higher doses of ropivacaine compared to the FNB/PSB group, accompanied by higher VAS scores. Patients’ satisfaction was equal between the groups. Both groups showed comparable mobilization rates and walking distances following TKA. **Conclusions:** IPACK/SACB showed equal results compared to FNB/PSB for mobilization rates and patients’ satisfaction following TKA without a reduction in opioid consumption.

## 1. Introduction

In patients with end-stage knee osteoarthritis, total knee arthroplasty (TKA) is the standard treatment, leading to pain relief, correction of deformity, and functional restoration [1]. Moderate to severe pain is common during the postop period and may lead to prolonged hospital stay, reduced quality of recovery, and higher use of opioids [2,3,4,5,6]. The widely used model of Enhanced Recovery after Surgery (ERAS) demands fully controlled postoperative pain to speed up patients’ recovery, shorten the hospital stay, and increase patient satisfaction [7,8]. Besides general anesthesia for TKA, surgery may also be performed under spinal anesthesia [9]. Nevertheless, spinal anesthesia does not provide any benefits for post-surgery pain management compared to general anesthesia, combined with a higher risk for hypotensive events [10,11]. Ultrasound-guided peripheral nerve blocks may help to reduce post-surgical pain without using opioids [1]. A femoral nerve block (FNB), in combination with a popliteal sciatic nerve block (PSB), offers reliable postoperative analgesia linked with relevant limitations like quadriceps femoris weakness, increased risk of falls, delayed rehabilitation, and nerve injuries [12]. Several studies have shown that a single adductor canal block (SACB) offers equal analgesic levels to a FNB without affecting the quadriceps femoris [13,14]. Unfortunately, this approach does not prevent the often-claimed posterior knee pain. Therefore, a SACB should be combined with a PSB or multimodal drug periarticular injections [1,15,16]. The ultrasound-guided infiltration between the popliteal artery and capsule of the posterior knee (IPACK) has been described in several studies as a promising analgesic procedure for posterior knee pain without muscular weakness and unwished injuries [15,17,18,19,20,21]. Several randomized control trials (RCTs), as well as a meta-analysis, have shown the impact of a SACB and IPACK for postoperative pain control and reduced opioid consumption [4,14,22,23], whereas one small study compared a SACB and IPACK with a FNB and PSB showing a mild impact on the early motor function in patients with a SACB and IPACK [24]. Moreover, one study compared the IPACK with the PSB, in which a benefit for early mobilization in patients with IPACK was observed [25]. Nevertheless, the described methods are discussed controversially, as most of the RCTs examined small cohorts. Moreover, one meta-analysis negotiated the effect of the IPACK when using surgeon-administered periarticular local infiltration analgesia (LIA) [26].

The aim of the study presented here was to evaluate patients with general anesthesia, using the IPACK combined with a SACB compared to an FNB combined with a PSB, in patients following primary unilateral TKA regarding postoperative pain levels, patients’ satisfaction and post-surgical mobilization.

## 2. Materials and Methods

### 2.1. Study Design

This is a retrospective cohort study investigating patients who received a unilateral total knee arthroplasty (TKA) for end-stage knee osteoarthritis at the orthopedic department at the Heidelberg University Hospital between 2021 and 2023. The study was approved by the local ethics committee (Ethics Committee of the Medical Faculty of Heidelberg, Trial Codes No. S-432/2023, date of vote: 16th August 2023, DRKS00033517) and conducted in accordance with the 1964 Helsinki Declaration and its later amendments. Clinical data collection was performed via IMI-EDC [27], an open-source electronic data capture tool developed by the Institute for Medical Informatics of the Heidelberg University Hospital in the orthopedic surgery department of the Heidelberg University Hospital. Due to the retrospective analysis of routine daily care data with anonymization of the data, no written informed consent was necessary and therefore not obtained. All routinely collected data, including demographics, procedure information, Visual analogue scale (VAS), and medication, were available for analysis. The study was performed according to the Strobe statement (Supplementary Statement S1).

### 2.2. Study Group Definitions

All patients with unilateral TKA (fixed-bearing, cruciate-retaining PFC Sigma (DePuy, Kirkel, Germany) or posterior-stabilized implant PFC PS (DePuy, Kirkel, Germany)) accompanied by peripheral regional anesthesia procedures in the observation period, independent from preexisting comorbidities, were included in the analysis. Patients were divided into two groups according to the peripheral nerve procedures used, which were always combined with general anesthesia. General anesthesia was performed in all patients by the use of propofol, sufentanil, and rocuronium dosed weight adapted. Maintenance of general anesthesia was obtained with sevoflurane; sufentanil was added when necessary. For postoperative analgesia, all patients received metamizole or paracetamol as a non-opioid as well as oxycodone, depending on their pain level, in accordance with a local predefined routine standard protocol.

The first group received preoperatively a combination of FNB and PSB, which was applied ultrasound-guided, as already described in the literature [24,28]. After detection of the femoral nerve, local cutaneous infiltration with 5 mL mepivacaine 1% was performed, and the needle was placed under ultrasound guidance. In case of negative aspiration, 20 mL ropivacaine 0.2% was injected, and the needle was removed. The same procedure was performed for the PSB after the detection of the sciatic nerve at the distal thigh.

The second group received a preoperative combination of IPACK and SACB, which was also applied ultrasound-guided, as already described in the literature [15,21]. In brief, the saphenous nerve was identified in the middle of the thigh for a SACB, local cutaneous infiltration with 5 mL mepivacaine 1% was performed, the needle was inserted ultrasound-guided, and 20 mL ropivacaine 0.5% was injected if aspiration was negative. For the IPACK, relevant structures were identified by ultrasound, local cutaneous infiltration with 5 mL mepivacaine 1% was performed, and the needle was placed. Following negative aspiration, 20 mL of ropivacaine 0.2% was injected, and the needle was removed.

Surgeon-administered periarticular local infiltration analgesia (LIA) with 50 to 100 mL ropivacaine 0.75% augmented with 500 μg adrenaline was administered, depending on the individual surgeon’s decision.

A primary total knee system was used in all patients. A cruciate-retaining TKR for knee osteoarthritis was performed by the implantation of the fixed-bearing, cruciate-retaining PFC Sigma (DePuy, Kirkel, Germany), as already described in [29]. Patients with an insufficiency of the posterior cruciate ligament (PCL) received a posterior-stabilized implant PFC PS (DePuy, Kirkel, Germany). Constrained condylar or rotating hinge knee arthroplasties were not included in the study cohort. A standardized operative technique with a midline incision, a medial parapatellar approach, and patellar eversion was used for TKR. The patella was selectively resurfaced when advanced degenerative changes with deep eburnation and grooving were seen intraoperatively. Components were fixed with cement (Refobacin bone cement R; Biomet, Berlin, Germany). Modified mechanical alignment was performed in all cases with the aim of achieving a straight leg axis in the coronal plane, whereas a slight mechanical (1° to 5°) postoperative varus/valgus alignment was tolerated in knees with severe preoperative varus/valgus deformities.

### 2.3. Outcome Parameters

Maximum VAS was registered in the Postanesthesia Care Unit (PACU) 24, 48, and 120 h after surgery. VAS at rest and at movement were recorded 24, 48, and 120 h post-surgery. Patients’ satisfaction was scored on a scale of 0 to 10, which indicates absolutely no satisfaction to absolute satisfaction at 24, 48, and 120 h post-surgery. Mobilization, walking distance, and range of knee motion with maximum knee extension in degrees and maximum flexion in degrees were entered in the database on days 24, 48, and 120 h post-surgery.

### 2.4. Statistical Analysis

All data were saved into an electronic database (Excel 365; Microsoft Corp, Redmond, WA, USA) and evaluated using SPSS software (Version 28.0; SPSS, Inc., Chicago, IL, USA). Figures were generated using GraphPad Prism 10 (GraphPad Software, La Jolla, CA, USA) and SPSS software and assembled with the presentation software PowerPoint 365 (Microsoft Corp, Redmond, WA, USA). Categorical data were shown as absolute and relative frequencies. Quantitative data were presented as median with quartiles. The Kolmogorov–Smirnov test was used to check for normal distribution. Due to non-normally distributed data, non-parametric methods for evaluation were used (chi-square test for categorical data, Mann–Whitney U test for continuous data). A *p*-value < 0.05 was considered statistically significant.

## 3. Results

### 3.1. Patient’s Characteristics

The patient’s characteristics are shown in Table 1. No significant differences were observed between the two groups regarding the demographic data. Patients with IPACK/SACB showed a significantly prolonged duration of stay at the Post Anesthesia Care Unit (PACU). Moreover, the time until the first treatment with opioids was shorter in the IPACK/SACB group compared to the FNB/PSB group despite a significantly higher rate of LIA application in the IPACK/SACB group.

### 3.2. Comparison of Pain Levels following Surgery

Patients with IPACK/SACB reported significantly higher pain levels in the PACU compared to patients with FNB/PSB (Table 2), accompanied by an increased need for opioids. Moreover, the time for the first requirement of opioids after PACU discharge was significantly shorter in the IPACK/SACB group. While pain levels at movement were comparable between the groups at 24 h and later time points, patients with IPACK/SACB reported significantly higher pain levels at rest and at 24 h and later on. The need for opioids was also increased in the IPACK/SACB group compared to the FNB/PSB group (Table 2), whereas the non-opioid analgetic drug doses used were comparable between both groups. A subgroup analysis showed no relevant differences in pain levels between patients with or without LIA (Supplementary Table S1).

### 3.3. Comparison of Mobilization, Muscle Strength, Degree of Movement, and Movement Distance

**Figure 1:**
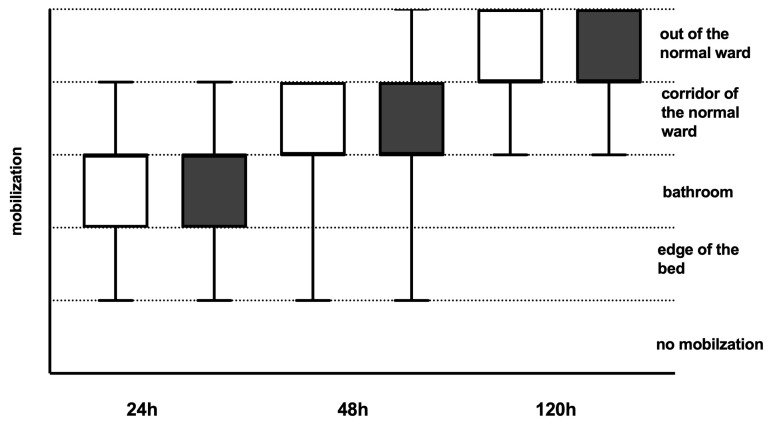
Mobilization rates of patients following total knee arthroplasty (TKA). Mobilization rates were recorded 24 h, 48 h, and 120 h following TKA and classified into 5 categories Dark grey boxes indicate patients with infiltration between the popliteal artery and capsule of the posterior knee (IPACK) and single adductor canal block (SACB) (*n* = 175), whereas white boxes indicate patients with femoral nerve block (FNB) and popliteal sciatic nerve block (PSB) (*n* = 167). Data presentation: box plots with median, 25th percentile, and 75th percentile in the box, as well as with the 10th and 90th percentiles at the end of the whiskers.

**Figure 2:**
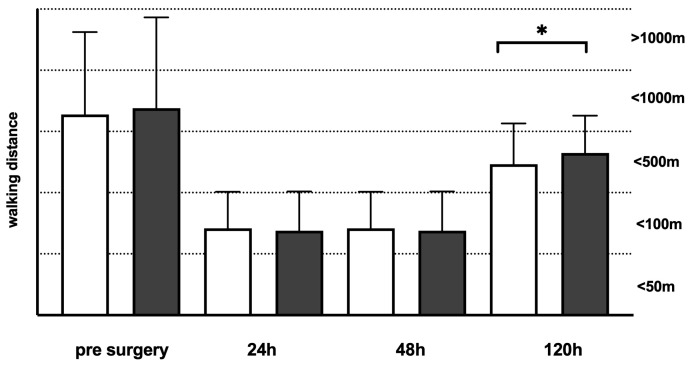
Walking distances of patients following total knee arthroplasty (TKA). Walking distances were recorded before surgery, 24 h, 48 h, and 120 h following TKA, and classified into 5 categories. Dark grey boxes indicate patients with infiltration between the popliteal artery and capsule of the posterior knee (IPACK) and single adductor canal block (SACB) (*n* = 175), whereas white boxes indicate patients with femoral nerve block (FNB) and popliteal sciatic nerve block (PSB) (*n* = 167). Data presentation: box plots with median, 25th percentile, and 75th percentile in the box, as well as with the 10th and 90th percentiles at the end of the whiskers. Symbols of significance: *p* < 0.05 *.

Both groups showed comparable mobilization rates within the first five days following TKA (Figure 1). Walking distances were longer before TKA and significantly longer on day 5 in the IPACK/SACB group compared to the FNB/PSB (Figure 2). Range of Motion (ROM) levels with regard to maximum knee extension in degrees and maximum flexion in degrees were significantly higher in the FNB/PSB group in the first two days following surgery. On day 5, the ROM levels showed comparable results (Table 3). No relevant reduction in muscle strength was observed in daily routine care in both groups. Patients with LIA showed a significantly reduced rate of flexion on the first two post-surgical days, normalizing on the following days without any clinical relevance (Supplementary Table S2).

### 3.4. Recorded Postoperative Complications

No complications like hematoma, paresthesia, or infections were reported in the IPACK/SACB group, whereas three patients in the FNB/PSB group claimed paresthesia during the application of the local anesthesia, which was not relevant in the post-surgical period. No relevant tendency to fall was expressed by all the patients.

## 4. Discussion

Within this retrospective analysis of clinical routine data, the combination of IPACK and SACB showed non-superior results for pain management in patients following TKA in comparison to FNB and PSB without relevant side effects or reduced opioid consumption.

Patients following TKA might suffer from severe pain in the early period following surgery. This, in turn, may lead to a reduced mobilization rate [2,3,4,5,6]. Several studies have shown the benefit of regional anesthesia by using single nerve blocks (e.g., FNB) or infiltration of tissue regions (IPACK, LIA) [1,12,13,14,15]. By the use of regional anesthesia procedures, reduced opioid consumption combined with increased movement rates might be reached, which is in line with the recommendations of the ERAS [7,8]. Apart from general anesthesia combined with regional anesthesia procedures, TKA may also be performed under spinal anesthesia [9]. Spinal anesthesia seems to possibly reduce the risk for non-home discharge compared to general anesthesia [9]. Nevertheless, spinal anesthesia does not provide improved post-surgery pain management and is linked with a high risk of hypotensive effects, which limits its usability [10,11].

Since its first description in 2012 by Sinsah, the positive effect of the IPACK has been described by several authors, which showed reduced opioid consumption, better movement rates, and a shortened length of hospital stay [13,17,19,20,21]. This effect might be increased by adding the SACB [4,14,22,23]. A relevant advantage of the IPACK seems to be the missing motor blockade, as no single nerve is the destination structure [21]. This, in turn, is comparable to our results, as no patients claimed muscle weakness or paresthesia. Nevertheless, we were not able to observe a reduced opioid consumption compared to patients with FNB/PSB. These findings are particularly in line with the findings of Texereia et al. [25], but in contrast to the results of Zheng et al., which described reduced opioid consumption by the use of IPACK [24]. Nevertheless, our findings might not be so controversial, as Zheng et al. examined only a small number of 60 patients [24] and most of the other studies compared IPACK/SACB to an opioid-based analgetic regime or a SACB [22,30,31,32,33,34], whereas Texereia et al. compared IPACK to PSB [25], which is, therefore, not fully comparable to our approach to compare IPACK/ SACB to FNB/PSB.

Moreover, even though our reported VAS scores showed statistically significant differences between the two groups at different time points, it should not be waived that the absolute pain levels are low in both groups and, therefore, mostly not relevant in daily routine clinical care.

Several studies describe muscular weakness in patients in the early post-surgery period following TKA as being caused by FNB or PSB [35,36]. Within our examined cohort, no muscular weakness was observed in patients with FNB/PSB, as the patients showed comparable movement or mobilization rates compared to patients with IPACK/SACB. In contrast to many of the published studies, which use ropivacaine 0.25–0.5% or carbostesine 0.25–0.5% [24,37,38,39], we used ropivacaine 0.2% to perform nerve block procedures. This might explain the missing muscular weakness due to low concentration and fluid volume. Moreover, our ultrasound-guided approach offered the potential to use reduced volumes of ropivacaine to reach comparable effects, which is in line with the literature [40].

Using LIA was described in the literature with reduced opioid consumption in patients following TKA, which might replace the IPACK [26]. Within our cohort, most of the patients with IPACK/SACB received LIA without a significant benefit in opioid consumption compared to patients with FNB/PSB. In line with this, we observed an increased volume of ropivacaine used in these patients combined with reduced flexion rates at the first two post-surgical days with normalization on comparable levels for patients without LIA, resulting in any clinical relevance in routine care for the single patient.

Zheng et al. described in their study a reduced muscular weakness in patients with IPACK/SACB compared to patients with FNB/PSB within the first 24 h following TKA [24]. Due to our retrospective study approach, no data were available describing muscular function or mobilization on the day of surgery. Therefore, no useful statement is possible out of our data. Nevertheless, the findings of Zheng et al. [24] are less relevant for our patients, as we start movement on day one following surgery and not on the day of surgery. Therefore, the effect of reduced opioid consumption predominates, especially while no imitated mobilization was observed in our examined cohort.

In Figure 1, we showed longer walking distances on day five in patients with IPACK/SACB compared to patients with FNB/SACB, which is in line with the findings of Zheng et al., Tang et al., and Guo et al. [23,24,41]. Nevertheless, this effect in our cohort might be caused by the fact that patients with IPACK/SACB had increased pre-surgery walking distances compared to patients with FNB/PSB, which offers a benefit for these patients in the post-surgery period.

In contrast to the walking distances, patients with FNB/PSB show significantly better ROM values compared to patients with IPACK/SACB. This is in contrast to several studies, which mostly compare FNB with SACB or continuous ACB [42], whereas Zheng et al. [24] do not report any data about ROM. Nevertheless, it has to be kept in mind that even though the reported values are significant, the differences are low between the two groups and may not be relevant in real clinical life.

### Limitations

The presented retrospective analysis of routine data is embossed by several limitations. First of all, the retrospective, single-center study design itself limits the meaningfulness of the data, even if the sample size of the included patients is large. Moreover, no detailed data about bromage and motor block were available, which limits the predictive value. Additionally, most of the patients with IPACK/SACB received LIA from the responsible surgeon, so its influence cannot be clearly assessed as a single parameter. Nevertheless, the presented data are real-world data from the clinical routine and, therefore, represent daily care reality, but they need to be proven in larger prospective trials.

## 5. Conclusions

IPACK/SACB showed equal results compared to FNB/PSB for mobilization rates and patients’ satisfaction following TKA without a reduction of opioid consumption, despite higher volumes of ropivacaine due to often combined LIA. By the use of low-concentrated ropivacaine, FNB/PSB might be an effective and safe pain control procedure following TKA without any relevant harm to patients’ mobilization or other complications.

## Figures and Tables

**Table 1:** Demographic characteristics and hospital data of patients undergoing total knee arthroplasty.

	All Patients *n* = 342	IPACK + SACB *n* = 175	FNB + PSB *n* = 167	*p*-Value
Age (years)	68.0 (61.0–74.8)	68 (61–75)	68 (61–74)	0.784
Gender (*n*, female/male)	220/122 (64.3/35.7)	110/65 (62.9/37.1)	110/57 (65.9/34.1)	0.513
BMI (kg/m^2^)	29.4 (25.9–33.8)	29.4 (26.2–34.9)	29.2 (25.8–32.8)	0.339
ASA grade				
I (*n*)	13 (3.8)	5 (2.9)	8 (4.8)	0.609
II (*n*)	188 (55.0)	100 (57.1)	88 (52.7)	0.609
III (*n*)	141 (41.2)	70 (40.0)	71 (42.5)	0.609
Smoker (*n*)	56 (16.4)	29 (16.6)	27 (16.2)	0.471
Alcohol habituation (*n*)	50 (14.6)	24 (13.7)	26 (15.6)	0.404
Chronic pain patient (*n*)	36 (10.5)	19 (10.8)	17 (10.2)	0.460
Duration of surgery (minutes)	100 (70–112)	100 (90–115)	100 (70–109)	0.430
LIA (*n*)	290 (84.8)	171 (97.7)	119 (71.2)	<0.01 **
Time to discharge to normal ward (minutes)	130 (100–165)	140 (115–175)	120 (94–150)	<0.01 **
Time to first treatment with opioids following surgery (minutes)	30 (10–62)	25 (5–51)	43 (5–75)	0.002 **
Hospital discharge (days)	7 (6–8)	8 (6–8)	7 (6–8)	0.354

Values are presented either as numbers (with the corresponding percentage values) or as median with accompanying quartiles (Q1:Q3). Legend: IPACK = infiltration between the popliteal artery and capsule of the posterior knee, SACB = single adductor canal block, FNB = femoral nerve block, PSB = popliteal sciatic nerve block, BMI = body mass index, ASA = American Society of Anesthesiologists, LIA = periarticular local infiltration analgesia. Concerning symbols and higher orders of significance: ** *p* < 0.01.

**Table 2:** Pain levels and satisfaction of patients undergoing total knee arthroplasty.

	All Patients *n* = 342	IPACK + SACB *n* = 175	FNB + PSB *n* = 167	*p*-Value
VAS pre-surgery	6 (5–7)	5 (4–6)	6 (5–8)	<0.001 ***
VAS post-surgery maximum	4 (2–6)	5 (3–6)	3 (0–5)	<0.001 ***
VAS 24 h maximum	5 (4–6)	5 (4–7)	5 (4–6)	0.429
VAS 48 h maximum	4 (3–5)	4 (4–5)	4 (3–6)	0.779
VAS 120 h maximum	3 (2–4)	3 (2–4)	3 (2–4)	0.035 *
VAS 24 h at rest	3 (2–4)	3 (2–4)	2 (2–3)	<0.001 ***
VAS 48 h at rest	2 (2–3)	2 (2–3)	2 (2–3)	0.039 *
VAS 120 h at rest	1 (1–2)	1 (1–2)	1 (0–2)	<0.001 ***
VAS 24 h at movement	5 (4–6)	5 (4–7)	5 (4–6)	0.416
VAS 48 h at movement	4 (3–5)	4 (4–5)	4 (3–6)	0.799
VAS 120 h at movement	3 (2–3)	3 (2–4)	3 (2–4)	0.035 *
Satisfaction 24 h	7 (6–7)	6 (6–7)	7 (6–7)	0.081
Satisfaction 48 h	7 (6–7)	7 (6–8)	7 (6–8)	<0.001 ***
Satisfaction 120 h	7 (6–8)	8 (7–9)	8 (7–9)	0.11
Morphine equivalent post-surgery	75 (0–165)	120 (75–195)	75 (0–105)	<0.001 ***
Morphine equivalent 24 h	45 (30–60)	45 (30–60)	37.5 (30–60)	0.066
Morphine equivalent 48 h	30 (30–45)	37.5 (30–45)	30 (30–45)	0.038 *
Morphine equivalent 120 h	15 (0–22.5)	15 (0–22.5)	7.5 (0–15)	0.039 *
Sufentanil intraoperative (μg)	40 (35–50)	45 (35–50)	40 (30–50)	<0.001 ***
Time until first opioid following PACU discharge (min)	660 (390–840)	600 (360–840)	720 (420–900)	0.022 *

Values are presented as median with accompanying quartiles (Q1:Q3). Legend: VAS = Visual analogue scale, IPACK = infiltration between the popliteal artery and capsule of the posterior knee (IPACK), SACB = single adductor canal block, FNB = femoral nerve block, PSB = popliteal sciatic nerve block. Concerning symbols and higher orders of significance: * *p* < 0.05, *** *p* > 0.001.

**Table 3:** Range of Motion (ROM) levels with regard to maximum knee extension in degrees and maximum flexion in degrees in patients undergoing total knee arthroplasty.

	All Patients *n* = 342	IPACK + SACB *n* = 175	FNB + PSB *n* = 167	*p*-Value
ROM before surgery	0 (0–0)/5 (0–10)/110 (100–120)	0 (0–0)/5 (0–10)/110 (100–120)	0 (0–0)/5 (0–10)/110 (100–120)	0.729
ROM at 24 h	0 (0–0)/0 (0–10)/75 (60–90)	0 (0–0)/5 (0–10)/70 (55–80)	0 (0–0)/0 (0–5)/80 (60–90)	0.007 **
ROM at 48 h	0 (0–0)/0 (0–10)/80 (70–90)	0 (0–0)/5 (0–10)/75 (60–90)	0 (0–0)/0 (0–5)/80 (70–90)	0.012
ROM at 120 h	0 (0–0)/0 (0–5)/90 (80–90)	0 (0–0)/0 (0–5)/90 (80–90)	0 (0–0)/0 (0–5)/90 (85–90)	0.256

Values are presented as median with accompanying quartiles (Q1:Q3). Legend: ROM = Range of Motion (ROM) levels with regard to maximum knee extension in degrees and maximum flexion in degrees, IPACK = infiltration between the popliteal artery and capsule of the posterior knee, SACB = single adductor canal block, FNB = femoral nerve block, PSB = popliteal sciatic nerve block. Concerning symbols and higher orders of significance: ** *p* < 0.01.

## Data Availability

The data presented in this study are available on request from the corresponding authors.

## Supplementary Materials

The following supporting information can be downloaded at: https://www.mdpi.com/article/10.3390/jcm13144079/s1, Table S1: Pain levels and satisfaction of patients undergoing total knee arthroplasty with regard on Surgeon-administered periarticular local infiltration analgesia (LIA); Table S2: Range of Motion (ROM) levels with regard on maximum knee extension in degrees and maximum flexion in degrees in patients undergoing total knee arthroplasty with focus on Surgeon-administered periarticular local infiltration analgesia (LIA); Statement S1: STROBE Statement—checklist.
